# Long-Term Follow-Up of Patients With Classic Fever of Unknown Origin: Prognostic Outcomes and Management Strategies

**DOI:** 10.1093/ofid/ofag372

**Published:** 2026-06-22

**Authors:** Li Zhang, Xiaotong Tian, BaoTong Zhou, Ying Ge, Hongwei Fan, Taisheng Li, Zhengyin Liu

**Affiliations:** Department of Infectious Disease, Peking Union Medical College Hospital, Chinese Academy of Medical Sciences, Peking Union Medical College, Beijing, China; Department of Infectious Disease, Peking Union Medical College Hospital, Chinese Academy of Medical Sciences, Peking Union Medical College, Beijing, China; Graduate School, Peking Union Medical College, Chinese Academy of Medical Sciences, Beijing, China; Department of Infectious Disease, Peking Union Medical College Hospital, Chinese Academy of Medical Sciences, Peking Union Medical College, Beijing, China; Department of Infectious Disease, Peking Union Medical College Hospital, Chinese Academy of Medical Sciences, Peking Union Medical College, Beijing, China; Department of Infectious Disease, Peking Union Medical College Hospital, Chinese Academy of Medical Sciences, Peking Union Medical College, Beijing, China; Department of Infectious Disease, Peking Union Medical College Hospital, Chinese Academy of Medical Sciences, Peking Union Medical College, Beijing, China; Department of Infectious Disease, Peking Union Medical College Hospital, Chinese Academy of Medical Sciences, Peking Union Medical College, Beijing, China

**Keywords:** clinical characteristics, diagnostic outcomes, fever of unknown origin, long-term follow-up

## Abstract

**Background:**

This study evaluates the long-term prognosis of patients with classic fever of unknown origin (FUO) who are discharged without a definitive diagnosis, enhances the understanding of FUO, and provides evidence-based guidance for its diagnosis and management.

**Method:**

A retrospective analysis was conducted on hospitalized patients at the Department of Infectious Diseases, Peking Union Medical College Hospital, who met the diagnostic criteria for classic FUO. Clinical characteristics and diagnostic outcomes were summarized, and patients without a definitive diagnosis (including those classified as clinical and indeterminate diagnoses) were examined longitudinally to determine their prognostic outcomes.

**Results:**

Overall, 739 patients with classic FUO were included. At discharge, 36.8% (*n* = 272) received a definitive etiologic diagnosis, 48.8% (*n* = 361) had a clinical diagnosis, and 14.3% (*n* = 106) remained indeterminate. Median hospitalization cost was significantly higher in the definitive diagnosis than in the clinical diagnosis group {22 000 RMB (interquartile range [IQR] 14 000–37 000) vs 17 000 RMB (IQR 11 000–29 000); *P* < .001}. Among 396 successfully followed-up patients discharged without a definitive diagnosis, mortality rate was 3.3% (13/396). During follow-up, 289 patients (73.0%) received a definitive diagnosis. The concordance rate between discharge and follow-up diagnoses was 93.0%, and the success rate of diagnostic antituberculosis therapy was 79.1% (53/67). Among the undiagnosed patients, 31.8% (34/107) experienced spontaneous remission, and 36.4% (39/107) achieved remission after short-term anti-inflammatory therapy.

**Conclusions:**

Most patients with classic FUO without a definitive diagnosis have a favorable prognosis, suggesting avoiding overtreatment. This study underscores the value of diagnostic therapies and close follow-up as key strategies in clinical management.

Fever of unknown origin (FUO) represents a heterogeneous group of diseases characterized primarily by prolonged fever. The concept was first formalized in 1961, when Petersdorf and Beeson defined it as a febrile illness lasting more than 3 weeks, during which the patient experienced recurrent fever continuously exceeding 38.3°C, but no definitive diagnosis could be established after at least 1 week of inpatient evaluation [1]. This definition was later classified as “classic” FUO to distinguish it from other FUO subtypes. In 2024, the International Fever and Inflammation of Unknown Origin Research Working Group proposed replacing the criterion for the evaluation duration with a minimal standard workup [2]. Despite greater consensus regarding its diagnostic criteria, FUO remains a diagnostic challenge worldwide. Approximately 7%–51% of patients with classic FUO do not receive a definitive diagnosis even after comprehensive assessment [3], with diagnostic efficiency varying according to population characteristics and regional healthcare standards.

More than 200 etiologies of classic FUO have been identified [4], broadly categorized as infectious, noninfectious inflammatory, neoplastic, and other diseases. Moreover, the diagnostic outcomes and etiologic distributions of FUO vary across time periods, regions, hospital tiers, and demographic groups [5–8]. When the etiology cannot be determined after a thorough systematic evaluation, clinicians often adopt empirical or diagnostic treatment strategies based on suggestive clinical manifestations or laboratory findings, for example, in suspected cases of tuberculosis (TB) or adult-onset Still's disease (AOSD). If the initial assessment fails to achieve a definitive diagnosis, it is advisable to refer patients promptly to specialists or multidisciplinary consultation teams to aid in identifying the underlying cause. A retrospective study [9] reported that among 192 patients referred to a tertiary center, 57.3% received a definitive diagnosis, while among the undiagnosed, approximately half improved after empirical therapy, with a mortality rate of 2.1%.

Generally, for FUO patients who remain undiagnosed despite extensive etiologic evaluation, follow-up studies conducted in recent years have been limited in number and sample size, providing insufficient long-term follow-up data on prognostic outcomes. Addressing this gap, the present study retrospectively analyzed the clinical records of inpatients with classic FUO at the Department of Infectious Diseases at Peking Union Medical College Hospital (PUMCH) and established a long-term follow-up cohort for patients without a definitive diagnosis (including those with clinical or indeterminate diagnoses). The goal was to enhance the understanding of FUO and provide evidence to inform optimized diagnostic strategies and stratified management approaches for classic FUO.

## METHODS

### Study Design and Patient Population

This retrospective cohort study included inpatients admitted for FUO at the Department of Infectious Diseases at PUMCH between December 2015 and November 2018. Patients who were discharged without a definitive diagnosis (including those with clinical or indeterminate diagnoses) were enrolled for long-term follow-up. Hospitalized patients who met the diagnostic criteria for classic FUO, as evaluated by senior attending physicians or higher-ranked specialists in the Department, were included in the study. However, the following patients were excluded:

1. Immunocompromised patients, defined as any of the following:
  - Neutropenia (absolute neutrophil count < 500 cells/μL) persisting for ≥1 week within the 3 months preceding fever onset
  - Uncontrolled human immunodeficiency virus (HIV) infection or CD4^+^ T-cell count < 200 cells/μL
  - Known hypogammaglobulinemia
  - Use of ≥10 mg/day prednisone (or equivalent dose of another corticosteroid) for at least 2 weeks within 3 months before fever onset
  - Receipt of immunosuppressive therapy for organ or hematopoietic stem-cell transplantation
  - Treatment with biologic agents, such as tumor necrosis factor (TNF) inhibitors or monoclonal antibodies
2. Incomplete or insufficient clinical data
3. Inaccessible information in the electronic medical record system
4. Any other condition that did not meet the study inclusion criteria

Data were extracted from the standardized inpatient electronic medical record system, including demographic characteristics, underlying diseases and relevant treatment history, and detailed clinical information, such as chief complaints, laboratory and imaging findings, length of hospital stay, hospitalization cost, discharge diagnosis, therapeutic regimen, postdischarge treatment, and prognostic outcomes.

### Definitions

Diagnostic criteria for classic FUO [1, 10]: Documented temperature > 38.3°C on multiple occasions, fever duration of ≥3 weeks, and no definitive diagnosis established after ≥1 week of comprehensive outpatient or inpatient evaluation and diagnostic testing.

Classification of diagnostic outcomes: Diagnostic outcome refers to the diagnostic status of each patient at hospital discharge.

Definitive diagnosis: This includes the fulfillment of any of the following criteria [11]: (1) meets established diagnostic criteria for a specific disease; (2) characteristic clinical manifestations plus positive histopathologic evidence (including bone marrow findings); (3) characteristic clinical manifestations plus positive microbiological evidence; (4) characteristic clinical manifestations plus both microbiological and imaging evidence; and (5) characteristic clinical manifestations plus 1 or more positive antigen or nucleic-acid tests for a pathogen, responsive to targeted anti-infective therapy.

Clinical diagnosis: No definitive etiologic confirmation; however, the clinical manifestations and test results indicate a probable disease; diagnostic clarification requires follow-up or assessment of therapeutic response.

Indeterminate diagnosis: Cases not meeting any of the above criteria, that is, a comprehensive evaluation reveals no probable etiology.

## DIAGNOSTIC CRITERIA FOR TUBERCULOSIS [12]

### Clinically Diagnosed Tuberculosis

Patients meeting all of the following criteria can be diagnosed with clinically suspected TB (ie, lacking direct microbiologic confirmation but a high clinical suspicion):

1. Presence of clinical symptoms (fever, fatigue, anorexia, night sweats, weight loss, cough, etc.) and radiological features suggestive of TB
2. Reasonable exclusion of other possible causes of FUO
3. At least 1 of the following supportive findings: moderate or strong positive tuberculin skin test (TST), positive interferon-γ release assay (IGRA), and histopathologic changes consistent with TB.

Tuberculosis is considered confirmed (active TB, ATB) when established by microbiological or histopathological evidence or when a clinically diagnosed case shows an effective response to diagnostic anti-TB treatment. When both pulmonary and extrapulmonary TB are present, the case is classified as extrapulmonary TB [13, 14].

### Confirmed Tuberculosis Cases

Confirmed TB is established when any of the following criteria are met:

1. Detection of *Mycobacterium tuberculosis* in body fluids or tissue by microbiological examination
2. Positive *M. tuberculosis*-specific DNA result by GeneXpert MTB/RIF assay on body fluid or tissue specimens;
3. Positive identification of *M. tuberculosis* by other molecular diagnostic methods (eg, line probe assay and real-time fluorescence PCR)
4. Histopathologic confirmation, defined as characteristic tuberculous granulomatous lesions (epithelioid cells and Langhans giant cells) observed in lung tissue, with positive acid-fast staining

### Follow-Up Management

Researchers conducted follow-up assessments of discharged patients and/or their family members via the electronic medical record system and telephone interviews to obtain postdischarge clinical status and management information for patients without a definitive diagnosis. Telephone follow-ups were performed between August 2024 and February 2025. Patient records were reviewed accordingly. Individuals who refused follow-up, could not be reached, or had no additional visit records in PUMCH's medical system were considered lost to follow-up.

### Statistical Analysis

All data were analyzed using SPSS Statistics version 29.0 (IBM, USA). Figures were generated using Excel, SPSS, or GraphPad Prism version 10.0 (GraphPad Software, USA). Continuous variables were tested for normality by conducting the Shapiro–Wilk test. Data conforming to a normal distribution were expressed as mean ± standard deviation (SD). Comparisons among 3 or more groups were performed using 1-way analysis of variance (ANOVA). Comparisons between two groups were performed by conducting an independent-samples *t*-test.

Non-normally distributed data were presented as median (interquartile range) (M [IQR]) and analyzed by conducting the Mann–Whitney U test (two groups) or the Kruskal–Wallis H test (3 or more groups). Categorical variables were summarized as frequency (*n*) and percentage (%), with between-group comparisons made by conducting a chi-square test or Fisher's exact test, as appropriate. All statistical tests were 2-tailed, with a significance level of *α* = .05 and *P* < .05 considered statistically significant.

## RESULTS

### Baseline Characteristics of the Study Population

**Figure 1.**
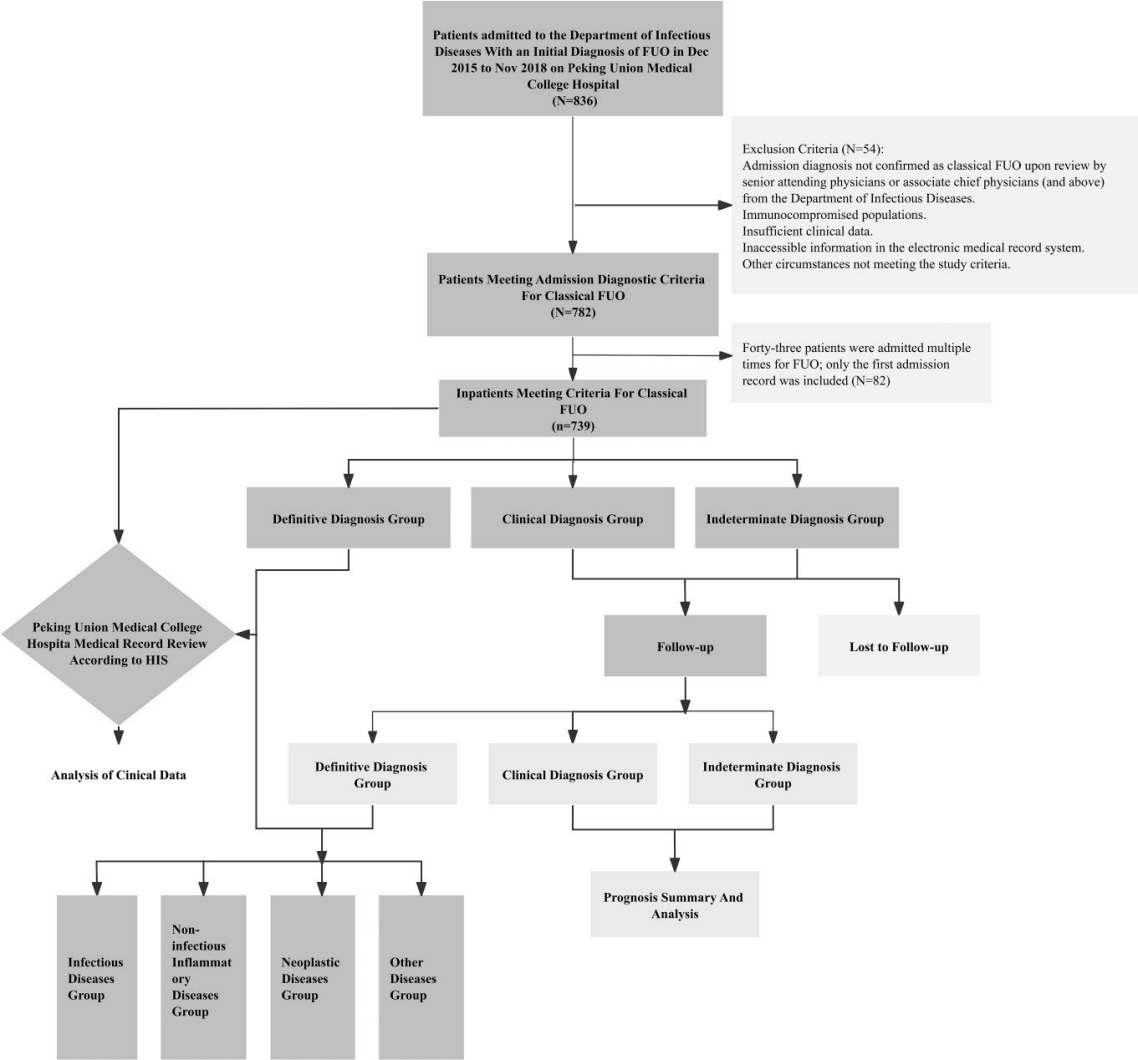
Technical roadmap of the study. Abbreviations: FUO, fever of unknown origin; HIS, Hospital Information System.

**Figure 2.**
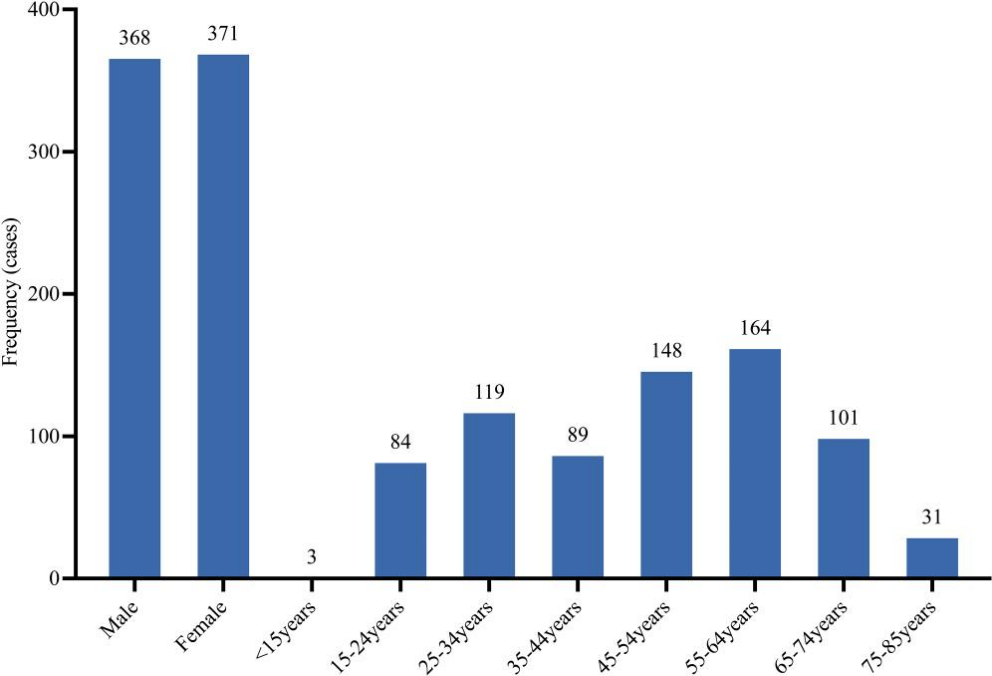
Sex and age distribution of patients with classic FUO admitted to the hospital between 2015 and 2018. Abbreviation: FUO, fever of unknown origin.

A total of 836 hospitalized patients with FUO were screened, and 739 patients who met the criteria for classic FUO were included in the study (Figure 1). Of these, 371 (50.2%) were female and 368 (49.8%) male. The median age was 50 years (IQR 32–61). According to age stratification, 607 patients (82.1%) were younger than 65 years, while 132 patients (17.9%) were aged 65 years or older (Figure 2).

### Diagnostic Outcomes at Discharge

Among 739 patients, 272 (36.8%) were assigned to the definitive diagnosis group, 361 (48.8%) to the clinical diagnosis group, and 106 (14.3%) to the indeterminate diagnosis group. In the definitive diagnosis group, the etiologic distribution was as follows: infectious diseases, 154 cases (56.6%); noninfectious inflammatory diseases, 54 cases (19.9%); neoplastic diseases, 27 cases (9.9%); and miscellaneous diseases, 37 cases (13.6%) (Table 1). In the clinical diagnosis group, the etiologic distribution was as follows: infectious diseases, 242 cases (67.0%); noninfectious inflammatory diseases, 94 cases (26.0%); neoplastic diseases, 19 cases (5.3%); and miscellaneous diseases, 6 cases (1.7%) (see Supplementary Figure 1 in Additional file 1).

**Table 1.** Number and Percentage of Cases by Etiology Among 272 Patients With Classic FUO

Etiologic Category	*N*	%
Infectious diseases	154	56.6
Bacterial infections	128	47.1
IE	24	8.8
TB	22	8.1
Brucellosis	18	6.6
Urinary tract infection	17	6.3
Pulmonary infection	16	5.9
Deep abscess	7	2.6
NTM infection	6	2.2
Biliary tract infection	5	1.8
Bone and joint infection	3	1.1
Bloodstream infection	2	0.7
Infectious aneurysm	2	0.7
Pyomyositis	2	0.7
Meningitis	1	0.4
Paratyphoid fever	1	0.4
Implant-associated infection	1	0.4
Soft-tissue infection	1	0.4
Viral infections	16	5.9
EBV	9	3.3
CMV	5	1.8
Meningoencephalitis	1	0.4
Hepatitis	1	0.4
Fungal infections	3	1.1
Aspergillosis	1	0.4
Candidiasis	1	0.4
*Penicillium marneffei* infection	1	0.4
Other infections	7	2.6
Mycoplasma infection	5	1.8
Legionella infection	1	0.4
Rickettsial infection	1	0.4
Noninfectious inflammatory diseases	54	19.9
Systemic vasculitis	15	5.5
PMR	6	2.2
SS	6	2.2
RA	5	1.8
AOSD	5	1.8
Undifferentiated CTD	3	1.1
Dermatomyositis	3	1.1
Reactive arthritis	2	0.7
SLE	2	0.7
BD	1	0.4
Undifferentiated arthritis	1	0.4
UC	1	0.4
RP	1	0.4
CRMO	1	0.4
RS3PE	1	0.4
PFAPA	1	0.4
Neoplastic diseases	27	9.9
Lymphoma	6	2.2
MDS	3	1.1
Castleman's disease	3	1.1
Carcinoma of the gastric cardia	2	0.7
Gastric cancer	2	0.7
Renal cancer	1	0.4
Prostate cancer	2	0.7
Lung cancer	1	0.4
Liver cancer	1	0.4
Colon cancer	1	0.4
Thyroid cancer	1	0.4
Metastatic cancer (primary site unknown)	3	1.1
Leiomyosarcoma	1	0.4
Other diseases	37	13.6
AA-PNH	1	0.4
DRESS	1	0.4
ECD	1	0.4
GD	2	0.7
HLH	4	1.5
Functional fever	3	1.1
HNL	11	4.0
AIN	2	0.7
ILD	4	1.5
Pheochromocytoma	1	0.4
Psychogenic fever	3	1.1
Subacute thyroiditis	4	1.5
Total	272	100

Abbreviations: AA-PNH, aplastic anemia-paroxysmal nocturnal hemoglobinuria syndrome; AIN, acute interstitial nephritis; AOSD, adult-onset Still's disease; BD, Behçet's disease; CMV, cytomegalovirus; CRMO, chronic recurrent multifocal osteomyelitis; CTD, connective tissue disease; DRESS, drug reaction with eosinophilia and systemic symptoms; EBV, Epstein–Barr virus; ECD, Erdheim–Chester disease; GD, Graves' disease; HLH, hemophagocytic lymphohistiocytosis; HNL, histiocytic necrotizing lymphadenitis; IE, infective endocarditis; ILD, interstitial lung disease; MDS, myelodysplastic syndrome; NTM, nontuberculous mycobacteria; PFAPA, periodic fever, aphthous stomatitis, pharyngitis, adenitis syndrome; PMR, polymyalgia rheumatica; RA, rheumatoid arthritis; RP, relapsing polychondritis; RS3PE, remitting seronegative symmetrical synovitis with pitting edema; SLE, systemic lupus erythematosus; SS, Sjögren's syndrome; TB, tuberculosis; UC, ulcerative colitis.

No statistically significant differences were observed among the 3 diagnostic-outcome groups based on sex, age, geographic region, educational level, marital status, medical bill payment option, lifestyle factors (smoking and alcohol history), comorbidities (hypertension, diabetes, or heart disease), and length of hospital stay (*P* > .05). For all patients with classic FUO, the median hospital stay was 23 days (IQR 15–32), and the median hospitalization cost was 20 000 RMB (IQR 12 000–31 000). However, hospitalization costs differed significantly among the diagnostic-outcome groups (*P* < .001). The definitive diagnosis group had a significantly higher median cost [22 000 RMB (IQR 14 000–37 000)] than the clinical diagnosis group [17 000 RMB (IQR 11 000–29 000); *P* < .001] (Table 2). No significant differences were observed among the groups regarding length of hospital stay or frequency of invasive procedures (*P* > .05) (Table 1).

**Table 2.** Demographic and Clinical Characteristics of Patients With Classic FUO by Diagnostic Outcome

Variable	Definitive Diagnosis (*n* = 272)	Clinical Diagnosis (*n* = 361)	Indeterminate Diagnosis (*n* = 106)	*P* Value
Demographic characteristics				
Male, *n* (%)	139 (51.1)	172 (47.6)	57 (53.8)	0.467
Age (years), M (IQR)	50 (32, 63)	49 (31, 61)	50 (35, 62)	0.560
Age (years), *n* (%)				
<65	215 (79)	304 (84.2)	88 (83)	0.236
≥65	57 (21)	57 (15.8)	18 (17)	
Region				
Urban	160 (58.8)	224 (62.0)	65 (61.3)	0.707
Rural	112 (41.2)	137 (38.0)	41 (38.7)	
Educational level, *n* (%)				
Illiterate	8 (2.9)	8 (2.2)	2 (1.9)	0.336
Primary school	31 (11.4)	40 (11.1)	13 (12.3)	
Junior high school	75 (27.6)	78 (21.6)	34 (32.1)	
High school	66 (24.3)	83 (23.0)	20 (18.9)	
College or above	92 (33.8)	152 (42.1)	37 (34.9)	
Marital status, *n* (%)				
Unmarried	44 (16.2)	51 (14.1)	15 (14.2)	0.924
Married	221 (81.3)	297 (82.3)	89 (84.0)	
Divorced	2 (0.7)	4 (1.1)	1 (0.9)	
Widowed	5 (1.8)	9 (2.5)	1 (0.9)	
Comorbidities, *n* (%)				
Hypertension	59 (21.7)	72 (19.9)	19 (17.9)	0.697
Diabetes mellitus *n* (%)	40 (14.7)	49 (13.6)	11 (10.4)	0.543
Heart disease *n* (%)	30 (11.0)	26 (7.2)	7 (6.6)	0.174
Clinical characteristics				
Length of hospital stay (days)	23 (16, 32)	24 (15, 33)	23 (14, 30)	0.542
Hospitalization cost (10 000 RMB)	2.2 (1.4, 3.7)	1.7 (1.1, 2.9)	2.0 (1.2, 2.9)	<0.001***
Invasive procedures, *n* (%)	191 (70.2)	257 (71.2)	85 (80.2)	0.130

Continuous variables are expressed as median (interquartile range) [M (IQR)]; categorical variables are presented as number (*n*) and percentage (%).

^***^ *P* < .001.

The proportion of patients admitted to the intensive care unit (ICU) differed significantly across the diagnostic-outcome groups (*P* = .003). The definitive diagnosis group had a higher ICU admission rate (3.7%) than the clinical diagnosis group (0.3%) and the indeterminate diagnosis group (0.9%). Pairwise comparison revealed a statistically significant difference in ICU admission rates between the definitive and clinical diagnosis groups (*P* = .0013).

### Long-Term Follow-Up Outcomes in Patients With Clinically Diagnosed or Indeterminate Classic Fever of Unknown Origin

**Figure 3.**
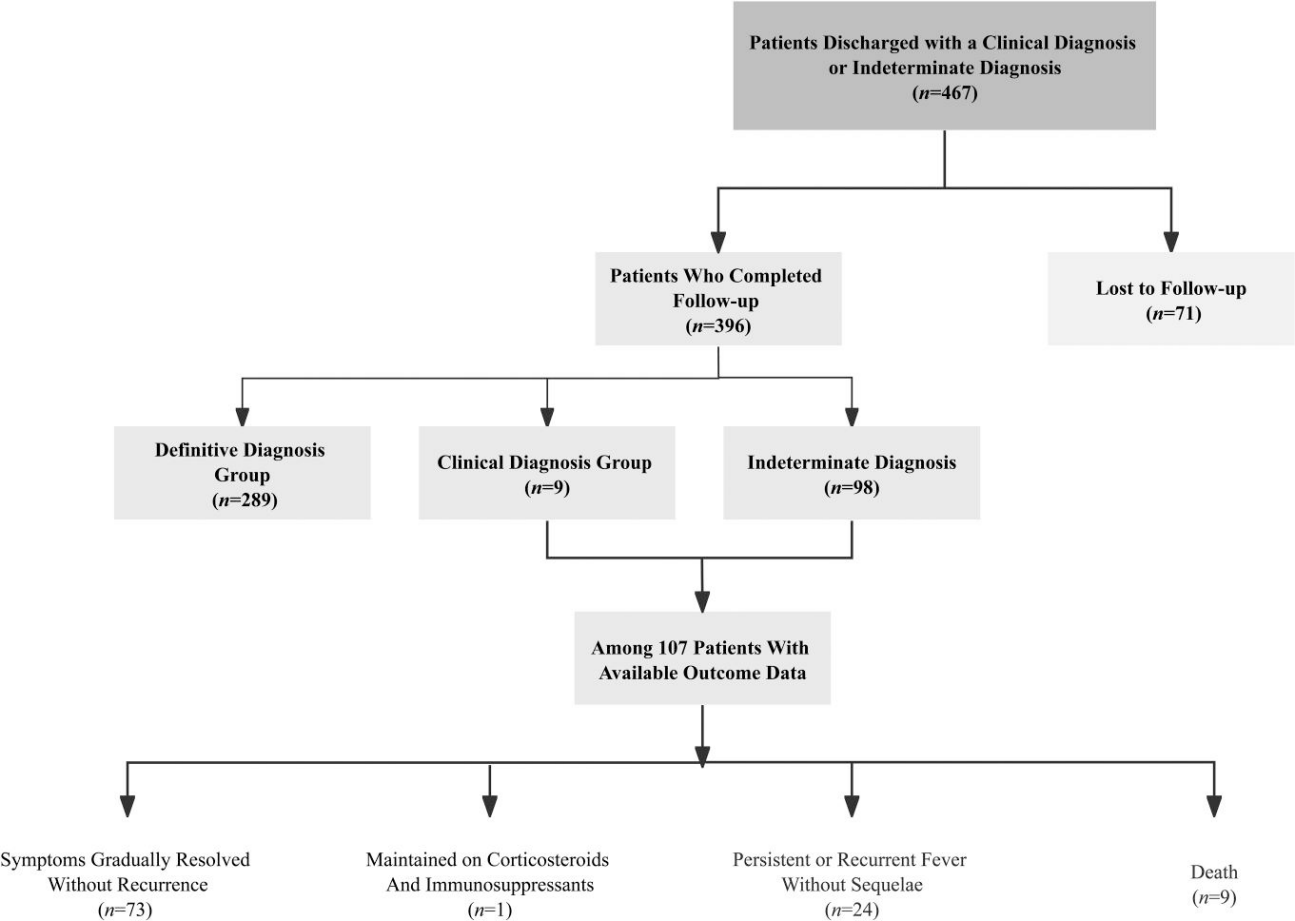
Technical roadmap for the follow-up of patients with clinically diagnosed and indeterminate FUO. Abbreviation: FUO, fever of unknown origin.

A total of 467 patients with classic FUO were discharged with either a clinical or indeterminate diagnosis. The median follow-up duration was 1836 days (IQR 213–2624). Follow-up was completed for 396 patients (84.8%) through a review of outpatient medical records or telephone contact, while 71 patients (15.2%) were lost due to lack of contact information or nonresponse. Among the patients followed, 194 (49.0%) were male and 202 (51.0%) female, with a median age of 49 years (IQR 31.5–60.0). During follow-up, 289 patients (73.0%) received a definitive etiologic diagnosis (see Supplementary Figure 2 in Additional file 1), while 107 patients (27.0%) remained within the clinical or indeterminate diagnosis categories (Figure 3).

Of the 361 patients discharged with a clinical diagnosis, 59 (16.3%) were lost to follow-up. Among the remaining 302 patients, 272 (90.1%) had a definitive diagnosis, 8 (2.6%) retained a clinical diagnosis, and 22 (7.3%) remained indeterminate.

Among patients with a definitive diagnosis, infectious diseases were most common (169/272, 62.1%), including viral infections (*n* = 113), tuberculosis (*n* = 53), and nontuberculous mycobacterial (NTM) infections (*n* = 3). Noninfectious inflammatory diseases accounted for 91 cases (33.5%), including AOSD (*n* = 46) and connective tissue diseases (CTD) (*n* = 36). The latter mainly comprised polymyalgia rheumatica (PMR) (*n* = 9), undifferentiated CTD (*n* = 9), and systemic vasculitis (*n* = 8), with fewer cases of Behçet's disease (BD) (*n* = 3), systemic lupus erythematosus (SLE) (*n* = 2), rheumatoid arthritis (RA) (*n* = 2), and single cases of Sjögren's syndrome (SS), inflammatory myopathy, and antisynthetase syndrome. Additional diagnoses included Crohn's disease (CD) (*n* = 2), ulcerative colitis (UC) (*n* = 1), relapsing polychondritis (RP) (*n* = 1), autoinflammatory diseases (*n* = 4) (with familial Mediterranean fever (FMF); periodic fever, aphthous stomatitis, pharyngitis, and adenitis (PFAPA) syndrome; synovitis–acne–pustulosis–hyperostosis–osteomyelitis (SAPHO) syndrome; and Schnitzler syndrome), and IgG4-related disease (*n* = 1).

Neoplastic diseases accounted for 11 cases (4.0%), including 10 lymphomas and 1 leukemia. One case (0.4%) was classified as miscellaneous and diagnosed as drug reaction with eosinophilia and systemic symptoms (DRESS).

The concordance rate between the discharge clinical and final follow-up diagnosis was 93.0% (253/272). These included infectious diseases, 167 cases (113 viral infections, 53 TB, and 1 NTM infection); noninfectious inflammatory diseases, 76 cases (41 AOSD, 28 CTD [6 PMR, 7 systemic vasculitis, 7 undifferentiated CTD, 2 SLE, 2 BD, 2 RA, 1 inflammatory myopathy, 1 antisynthetase syndrome], 2 CD, 1 UC, 3 autoinflammatory diseases [FMF, PFAPA, and Schnitzler syndrome], and 1 IgG4-RD); neoplastic diseases, 9 lymphoma cases; and miscellaneous disease, 1 DRESS (see Supplementary Figure 3 in Additional file 1).

Among the 124 patients discharged with a clinical diagnosis of viral infection, 113 improved rapidly during hospitalization or shortly after discharge, clinically confirming the diagnosis. These included 61 self-limiting cases, 40 treated with nonsteroidal anti-inflammatory drugs (NSAIDs), and 12 treated with short-term, low-dose corticosteroids. Eight patients were subsequently diagnosed with other conditions (3 CTD, 3 AOSD, 1 RP, and 1 leukemia), while 3 experienced recurrent or relapsing diseases.

Among 67 patients who received diagnostic or empirical anti-TB treatment and were followed up, 53 achieved successful outcomes (efficacy rate of 79.1%). Of these, 7 patients (13.2%) later obtained a confirmed microbiologic or pathologic diagnosis of TB, while 38 (71.7%) were extrapulmonary TB cases. The treatment failure group included 14 patients (20.9%), among whom 7 (10.5%) were eventually diagnosed with other diseases during follow-up (2 NTM infections, 1 AOSD, 3 PMR, and 1 giant cell arteritis [GCA]). Seven patients remained without a definitive diagnosis after follow-up.

Among the 106 patients discharged without a definitive diagnosis, 12 (11.3%) were lost to follow-up. Of the 94 patients successfully followed, 76 (80.8%) remained indeterminate, and 17 (18.1%) received a definitive diagnosis, all of which were noninfectious diseases. These included noninfectious inflammatory diseases—11 cases (64.7%), including 2 AOSD, 1 undifferentiated CTD, 1 systemic vasculitis, 1 large-vessel arteritis, 1 nodular nonsuppurative panniculitis, 2 NLRP12-associated autoinflammatory disease (NLRP12-AD), 1 SAPHO syndrome, 1 cryopyrin-associated periodic syndrome (CAPS), and 1 FMF; neoplastic diseases, 4 cases (23.5%), including 1 case each of indolent T-cell lymphoma, peripheral T-cell lymphoma, angioimmunoblastic T-cell lymphoma, and diffuse large B-cell lymphoma; and miscellaneous diseases, 2 cases, including 1 necrotizing lymphadenitis and 1 cold agglutinin disease.

### Prognostic Outcomes of Patients Remaining Undiagnosed at the End of Follow-Up

At the end of the follow-up, 107 patients (27.0%) remained in the clinical or indeterminate diagnosis categories. Among them, 34 cases were self-limited, 33 experienced symptom resolution after treatment with NSAIDs, 6 achieved remission following glucocorticoid (GC) therapy, and 1 required long-term GCs combined with immunosuppressive therapy for disease control. A total of 24 patients experienced persistent or recurrent fever for several months or even years after discharge, without developing other sequelae. Among these, 15 (62.5%) had self-limiting disease, 4 (16.7%) required intermittent treatment with GCs plus immunosuppressants, 3 (12.5%) required intermittent NSAID therapy, and 2 (8.3%) required intermittent GC therapy.

Among patients discharged with clinical or indeterminate diagnoses, 13 deaths were recorded, yielding a fatality rate of 3.3% (13/396). Of these, 4 deaths occurred in the definitive diagnosis group: 1 due to leukemia complicated by SARS-CoV-2 infection, 1 from respiratory failure secondary to pulmonary infiltration by mucosa-associated lymphoid tissue (MALT) lymphoma, 1 from AOSD complicated by severe pneumonia and septic shock, and 1 from infection-related septic shock following chemotherapy-induced neutropenia in diffuse large B-cell lymphoma (DLBCL). The remaining 9 deaths occurred in the clinical or indeterminate diagnosis groups, including 6 with suspected lymphoma, 1 with suspected metastatic carcinoma (primary site undetermined), 1 with suspected colorectal cancer complicated by hemorrhagic shock, and 1 with multisystem involvement, leading to multiple organ failure.

**Figure 4.**
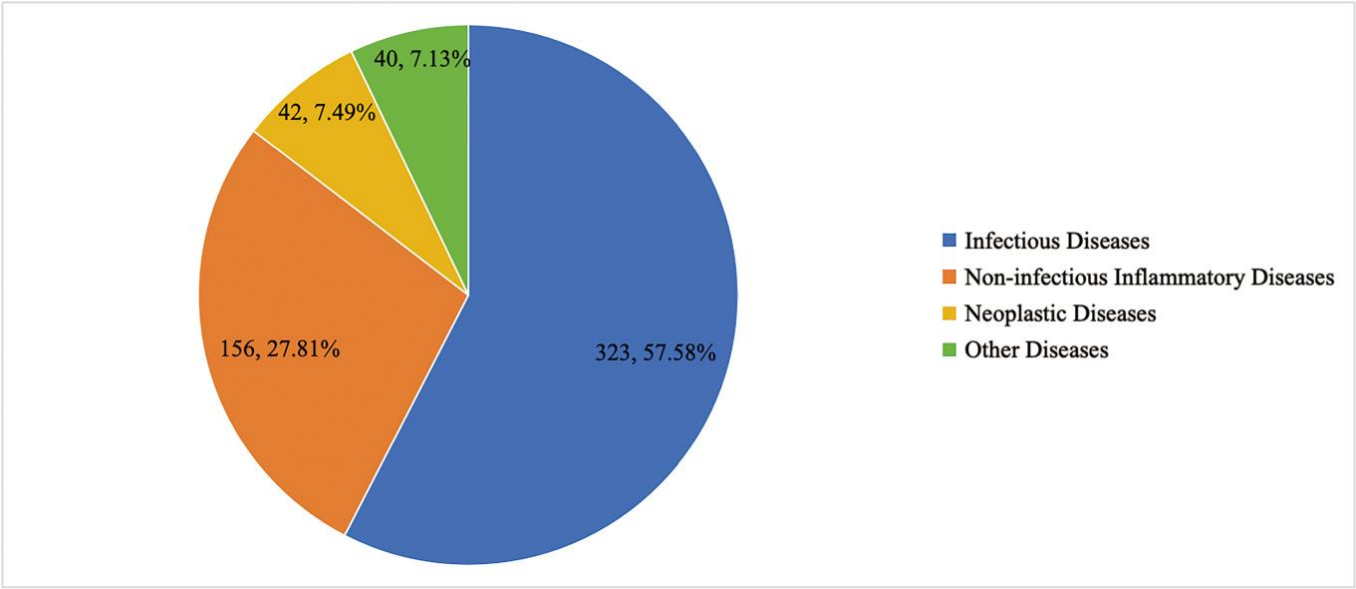
Etiologic distribution among 561 patients with definitive diagnoses established through systematic evaluation or follow-up.

After long-term follow-up, 561 patients received a definitive diagnosis. The etiologic distribution was as follows (Figure 4): infectious diseases, 323 cases (57.58%); noninfectious inflammatory diseases, 156 cases (27.81%); neoplastic diseases, 42 cases (7.49%); and miscellaneous diseases, 40 cases (7.13%).

## DISCUSSION

Classic FUO is a clinical syndrome characterized by atypical presentations of common diseases, involving complex etiologies and diagnostic challenges. At present, research examining the associations between clinical characteristics and diagnostic outcomes in classic FUO, as well as large-scale, long-term follow-up data for patients without a definitive diagnosis, is lacking. Therefore, this study is the first analysis of classic FUO cases managed at the Department of Infectious Diseases at PUMCH in China. It explored the associations between clinical characteristics and diagnostic outcomes and performed long-term follow-up of patients discharged without a definitive diagnosis to determine their prognoses, improving the understanding of classic FUO.

Among 739 patients in this study, the rates of definitive, clinical, and indeterminate diagnoses at discharge were 36.8%, 48.8%, and 14.3%, respectively. Compared with previous studies [2, 3, 6, 11, 15–17], the rate of indeterminate diagnosis in the cohort was slightly higher, likely due to the greater complexity of referred cases, heterogeneity of the patient population, and regional differences. However, when compared with data from 2004 to 2010 [18], the proportion of undiagnosed FUO cases was markedly decreased. Although advances in healthcare systems and the growing recognition of newly defined diseases have improved diagnostic capabilities, the overall etiologic spectrum of classic FUO remains largely consistent with previous reports [19–25].

Among the 272 patients with a definitive diagnosis, infectious diseases continued to represent the most common etiology (56.6%). Historical data from the Department showed a similar trend, with infectious diseases accounting for 51.3% of confirmed FUO cases during 1985–1989 [26], 56.8% during 2000–2003 [27], and 60.1% during 2004–2010 [18]. These findings indicate that, despite major advances in microbiologic testing technologies, the proportion of FUO cases attributed to infection has not declined significantly, suggesting that the diagnosis of atypical infectious diseases remains challenging.

Among bacterial infections, 18.8% (24/128) of cases were confirmed as infective endocarditis (IE). These patients required prolonged antimicrobial therapy, and several required cardiac surgery, partly explaining the higher ICU admission rate and greater hospitalization cost in the definitive diagnosis group. In the noninfectious inflammatory disease group, CTD was the most frequent cause (75.9%, 41/54), with a higher prevalence among female patients, consistent with previous studies [24, 28, 29]. However, AOSD accounted for only 9.3% of cases within the group, lower than in earlier reports (28.2% [11], 31.5% [18], and 67.7% [30]) but higher than reported by Koreli et al [28] (6.3%). As AOSD lacks specific clinical manifestations and is a diagnosis of exclusion [31], confirmation often requires extended follow-up and dynamic observation after discharge [32].

Although few long-term follow-up studies have examined patients with classic FUO who remain undiagnosed at discharge, with most studies being limited in scale, their conclusions are largely consistent. The vast majority of patients have favorable prognoses, and many exhibit spontaneous resolution of fever [3, 33–35]. This phenomenon may be associated with underlying immune regulatory abnormalities in such patients [36, 37].

A recent meta-analysis [2] reported that the spontaneous remission rate of classic FUO was approximately 20% (range 6%–45%), most commonly observed in viremia or early-stage autoinflammatory diseases with atypical clinical manifestations. In this cohort, 124 patients presented mild symptoms, and their fevers resolved spontaneously during hospitalization or shortly after symptomatic treatment with NSAIDs or short-term, low-dose GCs. Although these cases were clinically presumed to involve viral infection or postviral hyperinflammatory states, the possibility of immune-mediated injury leading to subsequent disease evolution during prolonged fever or hyperinflammation could not be excluded.

During follow-up, only 8 patients initially diagnosed with viral infection were later found to have discordant final diagnoses. For such patients, outpatient follow-up and dynamic observation after discharge were recommended to avoid unnecessary or excessive treatment. Previous research [37] has similarly suggested that after excluding infection and malignancy, empirical anti-inflammatory therapy with NSAIDs or GCs could improve prognosis and aid in diagnosis, particularly in AOSD cases. However, although empirical therapy can relieve symptoms, it carries an estimated 15% misdiagnosis rate [37] and may lead to false reassurance in self-limited illnesses. Therefore, treatment indications should be strictly defined.

In this study, the proportion of lymphomas among neoplastic diseases increased markedly from discharge to follow-up (86.7% vs 22.2%). A previous report [38] found that only 2 of 6 patients with suspected lymphoma were confirmed, whereas, in this cohort, 9 of 14 suspected lymphoma cases (64.3%) were verified. Among noninfectious inflammatory diseases, AOSD was the most common, accounting for 47.1% (48/102) of cases, of which 41 were concordant with their discharge diagnoses. Empirical therapy in these patients was generally well tolerated, with some patients achieving stable remission after treatment discontinuation. This aligns with the previously described “clinical–therapeutic response” diagnostic model [39]. Furthermore, among the 102 noninfectious inflammatory disease patients definitively diagnosed during long-term follow-up, 95.1% (97/102) were promptly referred to the Department of Rheumatology and Clinical Immunology at the institution. Of these, 9 patients (8.8%) were diagnosed with rare autoinflammatory diseases (AIDs), all characterized by recurrent fever and, in most cases, chronic disease courses. These included FMF (*n* = 2), PFAPA syndrome (*n* = 1), SAPHO syndrome (*n* = 2), Schnitzler syndrome (*n* = 1), NLRP12-AD (*n* = 2), and CAPS (*n* = 1). Therefore, patients with recurrent fever, elevated inflammatory markers, and consistently negative pathogen tests should be referred to an AID specialty clinic for comprehensive genetic testing [40] to facilitate early diagnosis and reduce the substantial psychological burden associated with repeated hospital visits.

Nevertheless, AIDs, which include monogenic and polygenic disorders [41], remain among the most diagnostically challenging causes of classic FUO. Genetic testing is costly and may yield negative results, even in clinically suspected cases. In the long-term follow-up cohort, 6 patients were clinically suspected of AIDs, half of whom underwent genetic testing, but the results were negative.

Beyond empirical anti-inflammatory therapy, diagnostic anti-TB treatment remains one of the most common clinical decisions among infectious causes of classic FUO. In this study, the diagnosis of TB in most cases was based on a comprehensive clinical assessment together with the response to diagnostic anti-TB therapy, rather than on treatment response alone. Previous studies [42, 43] have reported response rates of 60%–85% to diagnostic anti-TB therapy among patients with TB-associated classic FUO. Extrapulmonary TB frequently manifests as classic FUO; however, due to its insidious symptoms, variable clinical presentations, and nonspecific imaging findings [43], diagnostic delays are common. Moreover, TB is often difficult to differentiate from NTM infections, autoimmune diseases, or malignancies, making diagnosis particularly challenging [43–45]. In this study, the response rate to diagnostic anti-TB therapy was 79.1% (53/67), notably higher than the rate of initially confirmed TB cases at discharge. Among these, extrapulmonary TB accounted for 71.7% (38/53), while only 10.5% (4/38) had a documented history of TB or known exposure. By contrast, 50% (7/14) of patients who failed diagnostic anti-TB therapy were diagnosed with other diseases, including NTM infections, noninfectious inflammatory diseases, and lymphoma. These findings highlight the overlapping clinical features of TB, NTM infection, and noninfectious inflammatory diseases, emphasizing that clinicians must remain highly vigilant. Hence, during diagnostic therapeutic trials, close monitoring of treatment response and ongoing etiologic reassessment are essential. These findings should also be interpreted within the context of regional epidemiologic differences. The etiologic spectrum and diagnostic approach of classic FUO are strongly influenced by geographic disease prevalence. In TB-endemic regions, infectious diseases—particularly tuberculosis—remain a major cause of FUO, whereas noninfectious inflammatory diseases and malignancies are more commonly reported in low-TB-incidence countries, such as the United States. Given the global diversity in the prevalence of tuberculosis as a cause of FUO, healthcare resources, and clinical expertise, a universally standardized algorithm for classic FUO is not feasible. Instead, clinical judgment should prevail, and the decision to start empirical antituberculous therapy, among other treatments, must be tailored to the individual patient's presentation and the local epidemiological and healthcare context.

Reports on the mortality rate of classic FUO vary considerably across studies [35], with estimates ranging from 1.1% to 23.2%. In this cohort, 13 of 396 patients (3.3%) died from disease progression, representing a relatively low mortality rate. This favorable outcome may be attributed to long-term, structured follow-up and early, appropriate referral after discharge, allowing timely adjustment of diagnostic and therapeutic strategies. For patients with persistent or worsening symptoms, proactive efforts were made to facilitate early etiologic clarification or empirical intervention. Previous research [6] found that malignancy (47.5%) and uncontrolled severe infection (27.1%) were the leading causes of death among classic FUO patients. Consistent with those findings, malignant disease was the most frequent cause of death in the present study's cohort. Although the patient population was characterized by complex etiologies and a relatively high proportion of undiagnosed cases at discharge, the overall prognosis remained favorable.

This study has several limitations as follows. It was a single-center retrospective study. The study population was derived from a national referral center for complex and difficult cases, introducing selection bias. The loss to follow-up rate was 15.2%. For most patients, postdischarge clinical data were obtained via telephone interviews, lacking detailed documentation of subsequent management, which may have affected the granularity of outcome analysis. Nevertheless, the study featured a larger sample size and longer follow-up duration than previous studies, enhancing data reliability. Moreover, the findings confirmed that clinical decision-making and management strategies at discharge were appropriate for the vast majority of patients.

## CONCLUSIONS

The diagnostic outcomes of classic FUO are closely associated with disease severity, complexity, and clinical decision-making. For patients with mild or stable clinical manifestations, whose etiologies remain undetermined after comprehensive evaluation, long-term outpatient follow-up is appropriate and could help avoid unnecessary or excessive intervention. The prognosis of most classic FUO patients is favorable. When the initial evaluation provides strong diagnostic clues, empirical or diagnostic treatment may be attempted at an early stage. However, clinicians should carefully assess treatment responses and, when necessary, return to etiologic reassessment to ensure diagnostic accuracy. For patients presenting recurrent fever and persistently negative routine investigations, rare AIDs should be considered, and timely referral to a specialist is recommended for further evaluation and management.

## Supplementary Material

ofag372_Supplementary_Data

## Supplementary Data

Supplementary materials are available at *Open Forum Infectious Diseases* online. Consisting of data provided by the authors to benefit the reader, the posted materials are not copyedited and are the sole responsibility of the authors, so questions or comments should be addressed to the corresponding author.

## Notes

***Authors' contribution.*** All authors made a significant contribution to the work, whether in the conception, study design, execution, acquisition of data, analysis, and interpretation, or in all of these areas. They took part in drafting, revising, or critically reviewing the article, gave final approval for the version to be published, agreed on the target journal, and agreed to be accountable for all aspects of the work.

***Data availability.*** The datasets used and/or analyzed during the current study are available from the corresponding author upon reasonable request.

***Financial support***. This research was supported by the National High Level Hospital Clinical Research Funding (grant number 2022-PUMCH-B-043).
