# Supplementary material for: Long-Term Follow-Up of Patients With Classic Fever of Unknown Origin: Prognostic Outcomes and Management Strategies
Source: Open Forum Infect Dis. 2026 Jun 22;13(7):ofag372. doi: 10.1093/ofid/ofag372 (PMC13332407; doi:10.1093/ofid/ofag372)
Supplement: ofag372_Supplementary_Data [file ofag372_supplementary_data.zip › Supplementary Figures.docx]

**Supplementary Figure 1**. Distribution of etiologies among classic FUO patients, based on diagnostic outcomes

**Abbreviations:** FUO, fever of unknown origin

**Supplementary Figure 2**. Etiologic distribution of 289 patients with definitive diagnoses established during follow-up

**Abbreviations**: AOSD, adult-onset Still’s disease; CTD, connective tissue disease; CD, Crohn’s disease; UC, ulcerative colitis; RP, relapsing polychondritis; NLRP12-AD, NLRP12-associated autoinflammatory disease; FMF, familial Mediterranean fever; PFAPA, periodic fever, aphthous stomatitis, pharyngitis, and adenitis syndrome; SAPHO, synovitis-acne-pustulosis-hyperostosis-osteomyelitis syndrome; IgG4-RD, immunoglobulin G4-related disease; CAPS, cryopyrin-associated periodic syndrome; DRESS, drug reaction with eosinophilia and systemic symptoms


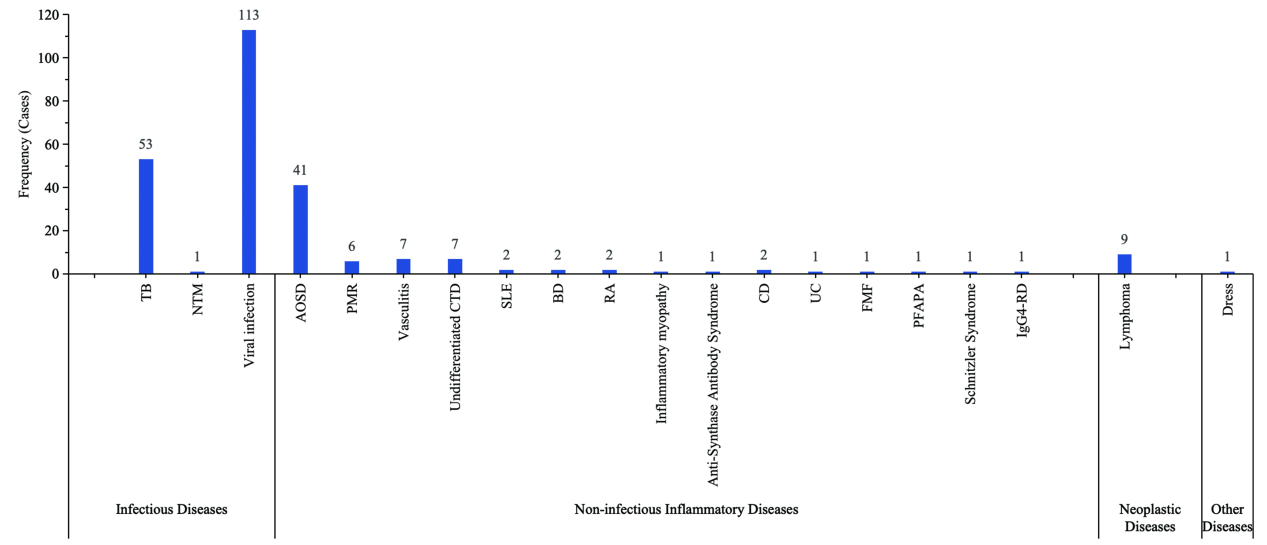


**Supplementary Figure 3**. Etiologic distribution of 253 patients whose follow-up diagnoses were consistent with their discharge clinical diagnoses

**Abbreviations**: AOSD, adult-onset Still’s disease; CTD, connective tissue disease; CD, Crohn’s disease; UC, ulcerative colitis; RP, relapsing polychondritis; NLRP12-AD, NLRP12-associated autoinflammatory disease; FMF, familial Mediterranean fever; PFAPA, periodic fever, aphthous stomatitis, pharyngitis, and adenitis syndrome; SAPHO, synovitis-acne-pustulosis-hyperostosis-osteomyelitis syndrome; IgG4-RD, immunoglobulin G4-related disease; CAPS, cryopyrin-associated periodic syndrome; DRESS, drug reaction with eosinophilia and systemic symptoms
